# Development and validation of the nutrition literacy scale for Chinese gout patients

**DOI:** 10.1371/journal.pone.0318259

**Published:** 2025-02-12

**Authors:** Wenjuan Zhang, Jiaqi Wang, Yan Wu, Yinglan Xia, Ziyu Sun, Yuhong Wu

**Affiliations:** 1 School of Nursing, Hangzhou Normal University, Hangzhou, China; 2 Zhejiang Greentown Cardiovascular Hospital, Hangzhou, China; Southern Medical University, CHINA

## Abstract

**Background:**

Nutrition literacy is essential for the self-management and treatment of gout patients. However, to date, there is no appropriate scale to measure the level of nutrition literacy gout patients in China.

**Objective:**

This study objective to develop and psychometric nutrition literacy scale for patients with gout.

**Methods:**

Using item development and psychological assessment. First, literature review, brainstorming, delphi study, and pr-survey were used to construct draft of the nutrition literacy scale for patients with gout. Second, 526 patients with gout underwent scale-based surveys. Item analysis and exploratory factors were used to optimize the scale. Confirmatory factor analysis was used to verify the structure of the scale, including the goodness of fit of the model, convergent validity, and discriminant validity. Subsequently, the reliability of the scale was tested using cronbach’s α coefficient and test-retest reliability.

**Results:**

The formal scale contains 5 dimensions: nutrition belief, nutrition knowledge, nutrition information acquisition ability, nutrition information interaction ability, nutrition information criticism ability, and 26 items. The overall content validity of the scale is 0.933. Exploratory factor analysis extracted 5 factors with a cumulative variance contribution of 82.18%. Confirmatory factor analysis showed that the scale structure was well fitted, with good convergent and discriminant validity. The overall cronbach’s α coefficient of this scale was 0.873, and the cronbach’s α coefficients of the dimensions were 0.861 ~ 0.980, and the overall re-test reliability was 0.864, and the re-test reliabilities of the dimensions were 0.881 ~ 0.981.

**Conclusion:**

Nutrition Literacy Scale for Gout Patients has good reliability and validity. It is suitable for the evaluation of nutrition literacy level in relevant population.

## Introduction

With the changes in living standards and dietary structure, the prevalence of adult hyperuricemia in China has reached 14% [1], making the incidence of gout is also on the rise, and according to epidemiological data, the global prevalence of gout has reached 15.30% [2], gout as a common disease is a serious threat to human health, the acute attacks of the disease caused by severe pain, activity limitation, as well as repeated hospitalization, which brings great physical and mental pressure to patients, and aggravates the economic burden of patients, and severely reduces the quality of life of patients [3]. Dietary management is the basis of gout treatment, and the correct adjustment of nutrition strategies can reduce the blood uric acid level of gout patients by 10%–18% [4], and reduce the number of gouty attacks, but the existing studies show that the adherence to dietary control of gout patients in China is only 37.8%, which is at a low level [5]. Nutrition literacy is the ability of individuals to access, process and understand basic nutrition information and services, and to make informed nutrition decisions, in 2015, Velardo expanded the theoretical framework of nutrition literacy based on the hierarchical model of health literacy [6,7], including that functional nutrition literacy refers to basic reading comprehension of nutrition-related information and the ability to master basic nutrition skills, which is the foundation of nutrition literacy; Interactive nutrition literacy refers to the ability to acquire nutrition information in the course of daily communication and activities, and to use the acquired nutrition information to make rational nutrition decisions; Critical nutrition literacy refers to the ability to critically analyse nutrition-related information and to address nutrition barriers, as well as an individual’s ability to judge the rightness or wrongness of nutrition information and to express a willingness to participate in societal nutrition activities. The knowledge-attitude-practice theory is classic [8] and overlaps with the hierarchical model of health literacy, where the knowledge aspect focuses on functional literacy, the behaviour aspect focuses on interactive and critical literacy, and the beliefs that form the basis of an individual’s behaviour should be added to the the hierarchical model of health literacy.

Nutrition literacy is closely related to patients’ dietary adherence, and studies have shown that nutrition literacy is a predictor of healthy eating behaviors in patients with chronic diseases [9,10]. Poor nutrition literacy may reduce an individual’s ability to make healthy dietary choices, resulting in individuals choosing poorer quality western diets, whereas good nutrition literacy leads individuals to make healthy dietary choices, such as a high quality mediterranean diet [10]. In addition, people with lower nutrition literacy are less likely to consult food labels, have more difficulty interpreting food labels and estimating appropriate food portion sizes, and have poorer diet quality [11,12].

Currently, the most widely used tool in the field of chronic diseases is the Nutrition Literacy Assessment Tool(NLAT) [13,14], which has been translated and culturally adapted by scientists from Spain, Italy, China and other countries, and has been validated and used in populations with diabetes mellitus, hyperlipidaemia, hypertension and overweight or obese people, However, this scale is a generalised scale and the dimensions of the scale are limited to the superficial knowledge level, which has not yet taken into account the willingness and interactive criticism level of patients to obtain information about diet and nutrition, and the scale items do not take into account disease-specific diet and nutrition-related content. Other specific scales, including the nutrition Literacy Measurement Tool for End-stage Renal Dialysis Patients(NLMTERDP) [15] and the Nutrition Literacy Scale for Peritoneal Dialysis Patients(NLSPDP) [16] have been validated and applied in China, and both were developed based on the hierarchical model of health literacy, which is more targeted, has good reliability and validity in the target population, and is able to accurately respond to the level of nutrition literacy in the measured population.

But there is no nutrition literacy assessment tool for patients with gout in the current study in China, so this study aims to develop an easy-to-understand, rapid, objectively measurable Nutrition Literacy Scale for Gout Patients(NLSGP) with a test of reliability and validity, in order to provide a scientific and effective measurement tool for the timely identification of poorly nourished patients and provide a scientific and valid assessment tool for timely intervention.

## Methods

### Study design

This study is a cross-sectional study. Using the knowledge, attitude and practice theory and the hierarchical model of health literacy as the theoretical framework to develop the NLSGP, followed by psychometrics to validate the reliability and validity of the scale.

### Sample and setting

Researcher using convenient sampling method, select gout patients from The Affiliated Hospital of HangZhou Normal University and Zhejiang Greentown Cardiovascular Disease Hospital from Jan 2024 to Jun 2024 for the survey. Recruitment took place in two phases: Round 1:Item analysis and exploratory factor analysis used for the scale. The sample size of factor analysis should be 5 ~ 10 times of the number of items of the scale [17]. The pretest version of the scale contains 28 items. Considering 10% invalid questionnaires, the sample size is 154–308 people, 246 gout patients were recruited in the first phase; Round 2:confirmatory factor analysis and reliability analysis for the scale. The sample size of confirmatory factor analysis should be more than 200 people, considering 10% invalid questionnaire [17], more than 220 people,280 gout patients were recruited in the second phase (excluding the first phase sample). Inclusion criteria: ① meet the diagnostic criteria for gout of the European League Against Rheumatism/American Rheumatism Association in 2015 [18]; ② age ≥ 18 years old; ③ have a certain level of verbal communication ability; ④ have no obvious cognitive dysfunction; ⑤ signed the informed consent form and have a certain degree of motivation for this study, and be able to complete the survey. Exclusion criteria: gout patients with combination of other critical diseases and unstable condition.

### Study procedures

**Fig 1:**
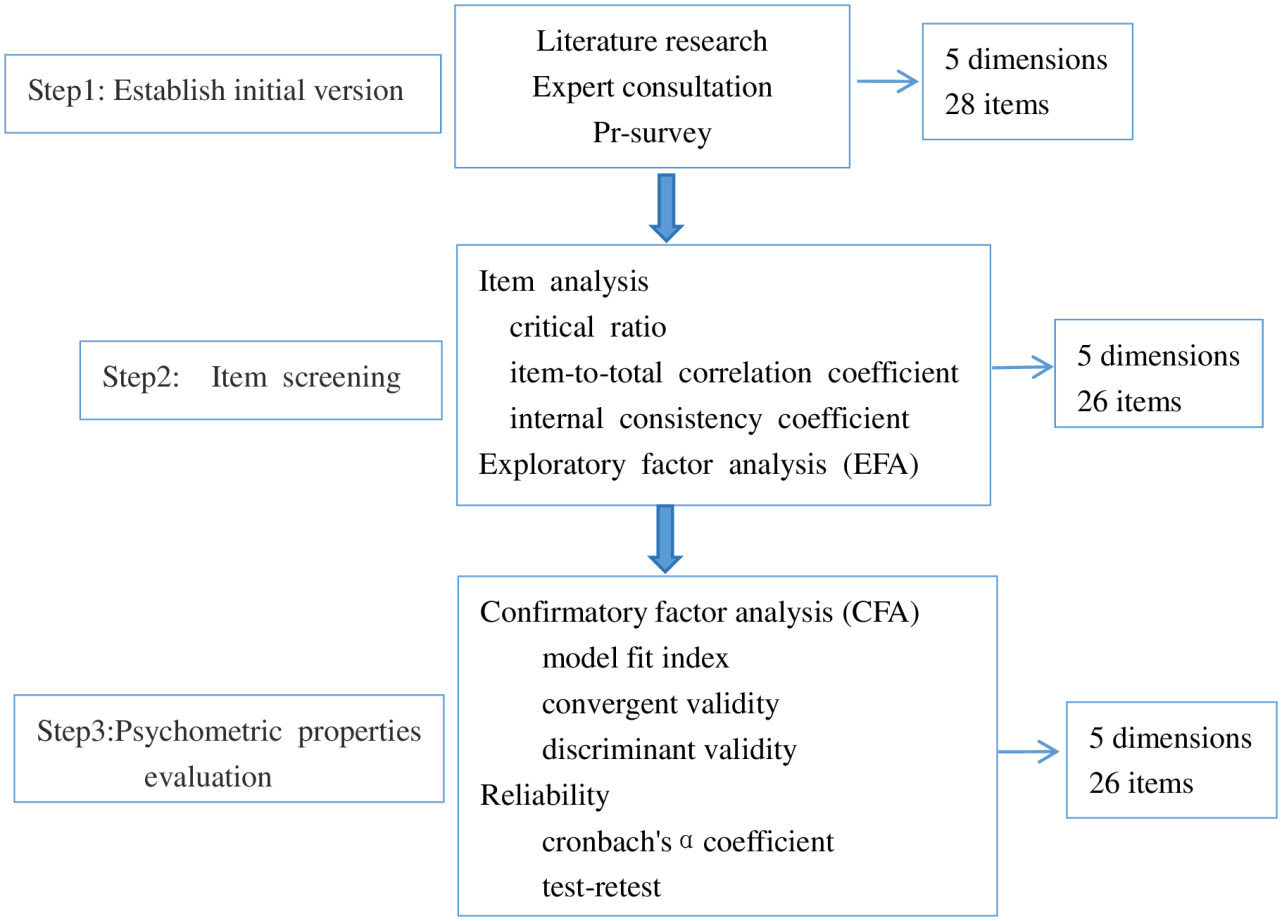
For Development of Nutrition Literacy Scale.

This study is divided into three steps: ① Establish initial version; ② Item screening; ③ Psychometric properties evaluation. See Fig 1.

#### Step 1: Establish initial version.

On the basis of the hierarchical model of health literacy [7] and the theory of knowledge, attitude and practice [8], we searched a number of databases(including:Pub Med, Web of Science, Embase, CINAHL, Scopus, Cochrane). The consensus of guidelines in relevant literature [19–21] was reviewed, combined with clinical practice and professional knowledge, and discussed with the research group members to construct the first draft of the NLSGP, which contained 33 items in 5 dimensions, among them, the nutrition belief dimension corresponds to the belief part in the knowledge, attitude and practice; the nutrition knowledge dimension corresponds to the functional health literacy part in the hierarchical model of health literacy;the nutrition information acquisition ability dimension and the nutrition information interaction ability dimension correspond to the interactive health literacy part in the hierarchical model of health literacy; and the nutrition information criticism ability dimension corresponds to the critical health literacy part in the hierarchical model of health literacy. The questionnaire was self-assessed and scored on a 5-point likert scale of “strongly disagree, disagree, not sure, agree, agree very much” or “never, occasionally, sometimes, often, always”, with scores of 1, 2, 3, 4, and 5.

We then conducted a delphi survey, inviting 12 experts from 5 provinces in China. They all held senior titles, had all worked for >20 years, and had expertise in medicine/chronic disease management/treatment, nutrition, medical humanities, or psychometrics. Experts rated the importance of each item and dimension using a 5-point Likert scale (ranging from 1 = not important to 5 = very important) and provided comments and suggestions on other items based on their experience and knowledge. Items were screened based on an importance score of ≥3.5 and a coefficient of variation of ≤.25 [22]. In the first round of expert consultation, the average importance scores of the items ranged from 3.50 to 5.00, and the coefficient of variation was 0 to .26. 9 experts gave their opinions, and after discussion, 3 items were deleted, 4 items were combined to form 2 items, and 11 items were modified. In the 2nd round of expert consultation, the mean score of importance of items was 4.08 to 5.00, and the coefficient of variation was 0 to .20. Experts were basically satisfied with the scale items, no deletion or addition, and only touching up some of the items, and the experts’ opinions tended to converge, so they ended the expert consultation, and after the discussion by the group, it was preliminarily determined that the scale contained 5 dimensions and 28 items.

Delphi survey used to assess the content of the scale validity, select expert enquiry for authoritative good 6 experts, evaluation scale includes enough, appropriate items, and scale items and subject is related, using Likert 4 grade score(1 is irrelevant, 2 divided into weak related, 3 is divided into strong correlation, 4 is very related). A content validity index (Item-CVI) ≥ 0.80 for each items versus a content validity index (Scale -CVI) ≥ 0.80 for the scale was used as the acceptance criterion result [23].

To test whether the presentation of the program was sufficiently clear to read and answer, a pr-survey was conducted in 24 gout patients, investigate the understanding of the content of the items and the difficulty of filling in the items, and record the problems and suggestions existing in the process of filling in. Revised the scale items by the investigator, and the pr-survey was undeleted and added items, forming the pretest version of the scale, which was still about 28 items in 5 dimensions.

#### Step 2: Item screening.

Item analysis:① the critical ratio method was used to evaluate the discriminatory nature of the items, and the total scores of the items were ranked from the highest to the lowest, obtaining the scores of the two groups of study subjects in the top 27.00% and the bottom 27.00% of the scores, comparing the scores of the items of the two groups, and retaining those with a decision value of ≥ 3.00 and *P* < .05 [24]. ② the correlation between the score of each item and the total scale score was evaluated using the correlation coefficient method, retaining the items with a correlation coefficient > .4.00 and *P* < .05 [24]. ③ the internal consistency coefficient method was used to test internal consistency, and if the Crombach’s α coefficient of the total table became significantly larger after deletion of an item, that item was deleted.

Exploratory factor analysis (EFA):We performed an exploratory factor analysis for the use SPSS26.0 software. Principal component analysis and maximum variance orthogonal rotation were used to retain dimension with ≥3 items and factor loadings ≥ .4.00 [25], under the common factor according to the principles of cumulative variance contribution > 60.00% and eigenvalue >1. Multiple loadings of the items with similar loading values were deleted (loadings were all >.4.00, and the difference was <.2.00).

#### Step 3: Psychometric properties evaluation.

AMOS 24.0 was used for validation factor analysis. The model fit indices are as follows: χ2/df < 3.00, root mean square error of approximation(RMSEA) < .08, normalized fit index(NFI), Tucker-Lewis index(TLI), Incremental Fit Index(IFI), comparative fit index(CFI) > .90, goodness of fit index (GFI) and adjusted goodness of fit index (AGFI) > .80 [26].

Convergent validity was evaluated by factor loadings, combined reliability (CR), and average variance extracted (AVE), and factor loadings greater than .50 [27]; AVE higher than .50 [28], and CR greater than .60 were also considered acceptable [29].

Discriminant validity was tested by comparing the AVE square root of each factor with the correlation coefficients between that factor and the other factors; if the AVE square root value was greater than the correlation coefficients, the scale was considered to have good discriminant validity [29].

Reliability was analyzed using the Cronbach’s α coefficient and test-retest reliability analysis, with a critical value of ≥ .70 for overall scale acceptability [30].

### Data collection

Before the investigation, the researcher will train the research team members to fill in the questionnaire, adopt the unified instruction to inform the purpose and significance of the study, and put the questionnaire to fill it in by themselves. For those with difficulty due to low vision and other reasons, the researcher will ask the offspring one by one.

### Ethical considerations

This study was approved by the Ethics Committee of the School of Nursing of Hangzhou Normal University (Approval No. 2023105). Informed consent was obtained from the patients after explaining the purpose, content, and method of the study. Patients are free to withdraw at any time. All information related to patient privacy was made anonymous and confidential.

## Results

### Sample characteristics

There were 526 people in total (Table 1). Among them 300 (57%) were female and 226 (43%) were male. Age was 41.15 ± 10.19 years. Living alone 95 (18.1%), living with spouse 257 (48.9), living with children 51(9.7%), living with spouse and children 123 (23.4%). Unmarried 12 (2.3%), married 420 (79.8%), divorced 53 (10.1%), widowed 41 (7.8%), etc.

**Table 1:** Participants’demographic data (n = 526).

Characteristic	n = 526
**Age, mean(SD)**	41.15 ± 10.19
**Gender**	
Male	226 (43%)
Female	300 (57%)
**Way of living**	
Live alone	95 (18.1%)
Living with spouse	257 (48.9%)
Living with children	51 (9.7%)
Living with spouse and children	123 (23.3%)
**Marital status**	
Unmarried	12 (2.3%)
Married	420 (79.8%)
Divorced	53 (10.1%)
Widowed	41 (7.8%)
**Residence**	
Rural	301 (57.2%)
City	225 (42.8%)
**Education level**	
Primary school and below	185 (35.2%)
Junior high school	100 (19.0%)
High school or junior college	118 (22.4%)
Colleges and above	123 (23.4%)

#### Step 1: Establish initial version.

The results show that the content validity index of the total table is 0.93, and the content validity coefficient of each item is 0.83 ~ 1.00, which shows that the content validity of the scale is good.

#### Step 2: Item screening.

Item analysis: ① critical ratio method:the values of the items ranged from 4.031 ~ 13.860, *P* < .05. ② the correlation coefficients of items C3 and E6 with the total score were less than.40 and were deleted; the correlation coefficients of the rest of the items with the total score ranged from .403 to .586, all *P* < .05. ③ internal consistency coefficient method: the results showed that the total Cronbach’s α coefficient of the scale was .877. The Cronbach’s α coefficient for the remaining items after deletion of each item did not exceed .877, ranging from .871 to .876. 26 items remained after item analysis (Table 2).

**Table 2:** The results of the item analysis (n = 246).

Items	Critical Ratio	Item-Total Correlation	Cronbach’s Alpha if Item Deleted	Reserved (R) or Deleted (D)
B1	13.860***	.579***	.871	R
B2	13.115***	.566***	.871	R
B3	12.096***	.573***	.871	R
B4	13.720***	.565***	.871	R
B5	12.560***	.567***	.871	R
B6	13.304***	.582***	.871	R
B7	13.197***	.585***	.871	R
B8	13.197***	.586***	.871	R
C1	6.939***	.425***	.874	R
C2	7.281***	.442***	.874	R
C3	6.390***	.386***	.875	D
C4	6.170***	.403***	.875	R
C5	7.128***	.432***	.874	R
D1	8.520***	.516***	.872	R
D2	10.532***	.569***	.871	R
D3	10.532***	.562***	.871	R
D4	9.845***	.535***	.872	R
D5	9.907***	.546***	.871	R
A1	6.482***	.430***	.876	R
A2	6.197***	.488***	.873	R
A3	4.151***	.438***	.876	R
A4	4.922***	.456***	.875	R
E1	7.773***	.488***	.873	R
E2	6.104***	.433***	.875	R
E3	5.970***	.468***	.874	R
E4	6.026***	.412***	.876	R
E5	5.495***	.436***	.875	R
E6	4.031***	.393***	.876	D
Criterion	≥3.000	≥.400	≤.877	

Note: ***P < .001.

Exploratory factor analysis: the results of exploratory factor analysis showed that Bartlett′s spherical test χ2 = 8789.911(*P* < .001) and KMO = .871, which was suitable for factor analysis. A total of 5 common factors were extracted, and the cumulative variance contribution rate was 82.18%%. The rotated matrix showed that the loadings of the items on the corresponding factors ranged from.741 to.963, all > .5, and a total of 26 items were retained (Table 3).

**Table 3:** Results of the exploratory factor analysis of the Nutrition Literacy Scale for Gout Patients (n = 246).

Items	1	2	3	4	5
B7.I know that I should eat fresh vegetables everyday.	0.946				
B2.I know that the consumption of red meat (pork, lamb, beef, etc.) should be controlled.	0.933				
B8.I know that I should drink at least 2000ml of water a day (including coffee and tea).	0.929				
B5.I know that crustacean seafood (lobster, shellfish, etc.) should be avoided.	0.919				
B6.I know that the intake of processed foods rich in fructose/sucrose (sweets, desserts, colas) should be controlled.	0.913				
B1.I know that animal offal (liver, kidneys, etc.) should be avoided.	0.907				
B3.I know that thick soups (rib soups, hot pot soups, etc.)should be avoided.	0.897				
B4.I know that I should avoid alcohol (including beer, wine, spirits, etc.) both on a daily basis and at parties and events.	0.896				
D3.After someone has explained the nutrition information, if I still don’t understand it, I will follow up with questions.		0.947			
D2.When I have a nutrition-related problem, I take the initiative to consult a professional.		0.942			
D4.I can clearly express my gout nutrition issues to others.		0.932			
D5.When I hear others have gout related nutrition issues, I proactively step forward to provide help and advice.		0.905			
D1.I communicated gout nutrition information with my family, friends, and patients.		0.905			
C5.I will look for gout diet information through official channels (hospitals, etc.).			0.963		
C4.I will look at gout books, newspapers, TV programmes, etc.			0.952		
C2.I will attend lectures on nutrition given by dietitians, doctors, nurses, etc.			0.942		
C1.I know where to get professional knowledge on gout dietary nutrition.			0.915		
E3.I will think about whether the gout diet information given to me by family and friends is correct.				0.858	
E2.I am going to think about whether the gout nutrition information in books, on TV and on the internet is scientific or not.				0.845	
E4.I will integrate the obtained dietary nutrition information and choose suitable foods based on my own dietary habits and preferences.				0.823	
E1.I will constantly adjust my diet to control my blood uric acid levels.				0.776	
E5.When faced with sales of dietary supplements and other products, I am able to make accurate judgments based on my own physical condition.				0.741	
A4.I am willing to change my dietary behavior to promote health.					0.829
A1.I think reasonable dietary control is beneficial for the development of gout.					0.828
A3.I am willing to learn more about gout nutrition.					0.787
A2.I think the purine content in the diet is related to the blood uric acid level in the human body.					0.753
Characteristic value	8.645	5.059	3.316	3.177	1.464
Cumulative variance contribution rate(%)	27.069	44.477	58.713	72.417	83.311

#### Step 3: Psychometric properties evaluation.

The results of the confirmatory factor analysis showed χ2/df = 1.122, RMSEA = .058, GFI = .911, AGFI = .892, CFI = .983, IFI = .983, TLI = .981, NFI = .916, The model fit criteria were met and the model fit was good (Table 4).

**Table 4:** Model goodness-of-fit (n = 280).

Index	Cutoff	Evaluation	
		Value	Rank
χ2/df	1–5	1.122	Good fit
RMSEA	≤.08	0.058	Good fit
GFI	≥.80	0.911	Good fit
AGFI	≥.80	0.892	Good fit
CFI	≥.90	0.983	Good fit
IFI	≥.90	0.983	Good fit
TLI	≥.90	0.981	Good fit
NFI	≥.90	0.916	Good fit

Note: χ^2^, Chi-squared; df, degrees of freedom; GFI, goodness-of-fit index; AGFI, adjusted GFI; RMSEA, root mean square error of approximation; NFI, normed-fit index; TLI, Tucker-Lewis index; CFI, comparative fix index; IFI, Incremental Fit Index.

The results of the convergent validity test showed that the standardised factor loadings for each item ranged from .652 to .902. The average variance extracted (AVE) values of the four latent variables ranged from .514 to .781, all of which were greater than .50, and their respective combined reliability (CR) values ranged from .816 to .935, all of which were greater than.60, suggesting that the assessment tool has good convergent validity (Table 5).

**Table 5:** Results of the convergent validity test (n = 280).

Items	Latent variable	Standardized factor loading	AVE	CR
A1	Nutrition belief	.764	.527	.816
A2		.729		
A3		.683		
A4		.724		
B1	Nutrition knowledge	.804	.514	.894
B2		.674		
B3		.679		
B4		.794		
B5		.762		
B6		.685		
B7		.669		
B8		.652		
C1	Nutrition information acquisition ability	.855	.781	.935
C2		.880		
C4		.902		
C5		.898		
D1	Nutrition information interaction ability	.872	.586	.875
D2		.673		
D3		.773		
D4		.791		
D5		.701		
E1	Nutrition information criticism ability	.732	.567	.867
E2		.708		
E3		.772		
E4		.838		
E5		.706		

Note: AVE, average variance extracted. CR, construct reliability.

The results show that the arithmetic square root values of AVE for the five latent variables are.717 ~ .884 which are all greater than the correlation coefficients with the other latent variables, indicating that there is good discriminant validity between the observational indicators of the different latent variables and that the assessment tool has good discriminant validity (Table 6).

**Table 6:** Results of the discriminant validity test (n = 280).

Latent variable	Nutrition information criticism ability	Nutrition information interaction ability	Nutrition information acquisition ability	Nutrition knowledge	Nutrition belief
Nutrition information criticism ability	**0.884**				
Nutrition information interaction ability	0.499	**0.753**			
Nutrition information acquisition ability	0.065	0.044	**0.765**		
Nutrition knowledge	0.503	0.450	0.074	**0.717**	
Nutrition belief	0.517	0.523	0.128	0.639	**0.726**

Note. The bold part is the the square root of AVE.

The Cronbach’s α coefficient for the formal scale was 0.873, and the Cronbach’s α coefficient for the dimensions of nutrition belief, nutrition knowledge, nutrition information acquisition ability, nutrition information interaction ability, nutrition information criticism ability were 0.861, 0.980, 0.972, 0.972, and 0.894, which indicated that there was a good internal consistency reliability of the scale; the re-test reliability of the scale was 0.864, and the re-test reliability of the dimensions of nutrition belief, nutrition knowledge, nutrition information acquisition ability, nutrition information interaction ability, nutrition information criticism ability were 0.881, 0.975, 0.973, 0.981, and 0.911, indicating that the scale has good external stability (Table 7).

**Table 7:** Results of the reliability analysis results(n = 280).

Latent variable	Cronbach’s α coefficient	Test-retest
Overall Scale	0.873	0.864
Nutrition belief	0.861	0.881
Nutrition knowledge	0.980	0.975
Ability to acquire nutrition information	0.972	0.973
Ability to interact with nutrition information	0.972	0.981
Ability to critically appraise nutrition information	0.894	0.911

### Final instrument

Based on the results of the item analysis, reliability and validity analyses, NLSGP consists of 26 items in 5 dimensions: nutrition belief (4), nutrition knowledge (8), nutrition information acquisition ability (4), nutrition information interaction ability (5), and nutrition information criticism ability (5). The questionnaire was self-assessed and scored on a 5-point likert scale of “strongly disagree, disagree, not sure, agree, agree very much” or “never, occasionally, sometimes, often, always”, with scores of 1, 2, 3, 4, and 5. The higher the total score, the higher the level of nutrition literacy.

## Discussion

In this study, an extensive literature search was conducted and the initial items of the scale were constructed using existing nutrition literacy assessment tools. 12 experts in related fields were selected from different provinces for 2 rounds of expert consultation, covering clinical medicine, nursing, nutrition and other fields representative in terms of geography and specialisation. Based on the experts’ opinions and the results of the consultation, the research group held several rounds of discussion and made changes to the scale items. In the survey phase, 24 gout patients were selected for a pr-survey to ensure the feasibility of the scale. The whole process of scale development is rigorous, reasonable in structure, clear in presentation and has a degree of scientific validity.

Bartlett′s spherical test χ2 = 8789.911 (*P* < .001) and the KMO = .871, which was suitable for exploratory factor analysis. Five common factors with eigenvalues > 1 were extracted, and the cumulative variance contribution rate was 82.18%, which was much higher than that of other nutrition literacy scales 56.31% (Kidney Scale [15]) and 61.82% (Dialysis Scale [16]). The loadings of each item in the corresponding factor ranged from.741 to.963, all of which were > .50, and the rotated factor loading matrices were basically consistent with the theoretical framework of scale development, indicating that the scale structure was reasonable. It was further verified by a validated factor analysis. After modifying the model, the factor loadings of each item ranged from .652 to .902, the χ2/df was 1.122, the RMSEA was .058, and the IFI, TLI and CFI were all > .90, indicating that the overall fit of the scale was ideal. The model AVE values ranged from .514 to .781, and the combined reliability values ranged from.816 to.935, indicating that the aggregated validity of the scale was good. The square root of the AVE of each factor was greater than the correlation coefficient between the factor and other factors, indicating that the scale had good discriminant validity. The final I-CVI of this scale ranged from .83 to 1.00 and the S-CVI was .93, indicating that the scale has good content validity. The overall cronbach’s α coefficient of this scale was .87, and the cronbach’s α coefficients of the dimensions were .86 ~ .98, and the overall re-test reliability was .86, and the re-test reliabilities of the dimensions were .88 ~ .98, which were within the acceptable range, proving that this scale has good reliability.

The original chronic disease pervasive scale [14] only includes a functional nutrition literacy component that focuses on assessing patients’ nutrition knowledge. In this study, we constructed a nutrition literacy scale based on the health literacy hierarchical model, the knowledge, attitude and practice Theory, and combined with the characteristics of gout patients in their daily dietary management, in order to expand the measurement range of nutrition literacy, with a higher degree of specificity, and focusing on gout patients. The final scale contains 5 dimensions and 26 items, including nutrition belief, nutrition knowledge, nutrition information acquisition ability, nutrition information interaction ability, and nutrition information criticism ability.

Nutrition belief are patients’ perceptions of the benefits and potential harms associated with nutrition knowledge and dietary management according to medical advice, and beliefs are the basis from which individual behaviours emerge, which implies that patients’ own nutrition cognition and attitude, as an intrinsic motivation, can drive them to consciously pay attention to and learn about relevant nutrition knowledge, and so on. Consistent with the results of similar studies [31,32], patients’ cognitive belief largely determine patients’ behaviour, and the level of dietary management is positively correlated with patients’ own cognition.

Nutrition knowledge reflects that patients should have adequate nutrition knowledge and be able to understand and comprehend nutrition information. Gout patients need to control the intake of high-purine foods over a long period of time and balance all types of nutrients. However, previous studies have shown [33] that patients have a vague and one-sided understanding of high-purine foods in their daily lives, and that misunderstanding of nutrition knowledge leads to unstable blood uric acid levels. Therefore, health professionals need to further improve the form of health education and optimise the nutrition knowledge in the guidelines to develop an educational manual that is easy for patients to understand [34], and the eight items established in this study are basic and important knowledge that can be used as a reference for the clinic.

Nutrition information acquisition ability refers to the ability of patients to access nutrition information in various forms, including the internet, books and lectures. Nutrition information interaction ability refers to the ability of patients to clearly express their needs when communicating with others and to receive help from others. The results of previous studies have shown [35] that patients will communicate with their family, friends and professionals to share their confusion and experiences in the process of dietary management, and will be able to receive emotional support, psychological comfort and professional support from their family, peers and professionals, and in this study, the settings of “D4. I can clearly express my gout nutrition issues to others.” and “C2. I will attend lectures on nutrition given by dietitians, doctors, nurses etc” can better reflect the above contents.

Nutrition information criticism ability refers to the ability of patients to recognise the accuracy, applicability and reliability of nutrition information from different sources and to make appropriate nutrition decisions in the light of their own situation. Consistent with similar studies [36], patients’ ability to critically analyse different types of health information facilitates patients’ disease management and thus improves disease prognosis. Therefore this study was designed with relevant entries to measure the level of critical nutrition literacy, for example,“E3. I will think about whether the gout diet information given to me by family and friends is correct.”“E4. I will integrate the obtained dietary nutrition information and choose suitable foods based on my own dietary habits and preferences.”

In conclusion, the scale constructed in this study includes not only the nutrition knowledge, beliefs, behaviours and skills of individuals, but also the social aspects such as family and medical support, thus highlighting the nutrition characteristics of gout patients in a comprehensive and specific manner.

## Limitations

There are some limitations to this study: First, the study did not assess criterion validity due to the lack of a gold standard for assessing nutrition literacy in gout patients. Second, this study used convenience sampling method, the sample was limited to Hangzhou city, China, and the patients were younger, which made the sample less representative, and may affect the generalization and application of the scale. Finally, due to time constraints, we did not use the developed scale to assess the level of nutrition literacy in gout patients.

## Conclusion

The Nutrition Literacy Scale for Gout Patients compiled in this study strictly followed the scale development process, resulting in 26 items in 5 dimensions with good reliability and validity, which can be used as a measurement tool to assess the nutrition literacy of gout patients. In the future, firstly, researchers should conduct multi-centre and large-sample studies to expand the scope of the scale, further determine the optimal cut-off score (e.g., what is the poor level, what is the moderate level, and what is the high level) of the Nutrition Literacy Scale for Gout Patients, which is more meaningful for the promotion and clinical use of the scale. Secondly, the mean age of the patients in this study was 41.15 ± 10.19, which requires further validation in the elderly population. Thirdly, the content of the scale items can be further strengthened. Many chinese believe in chinese medicine and use it extensively in their daily lives, and it is also used in the treatment of many diseases [37], so the scale can be improved by including knowledge related to nutrition in the items. In addition, the researchers can use this scale to investigate the nutrition literacy of gout patients and fully explore its influencing factors, including the use of drugs and comorbidities.

## Supporting information

S1 File Dataset. (XLSX)
